# Theoretical Framework for Plastic Waste Management in Ghana through Extended Producer Responsibility: Case of Sachet Water Waste

**DOI:** 10.3390/ijerph120809907

**Published:** 2015-08-20

**Authors:** Ebo Tawiah Quartey, Hero Tosefa, Kwasi Asare Baffour Danquah, Ilona Obrsalova

**Affiliations:** 1 Faculty of Chemical Technology, Institute of Environmental and Chemical Engineering, University of Pardubice, Pardubice 573, 53210, Czech Republic; 2 Faculty of Economics and Administration, Institute of Administrative and Social Sciences, University of Pardubice, Pardubice, 95, 532 10, Czech Republic, E-Mail: ilona.obrsalova@upce.cz

**Keywords:** plastic waste management, producer responsibility, recovery, sachet water waste, environmental problems

## Abstract

Currently, use and disposal of plastic by consumers through waste management activities in Ghana not only creates environmental problems, but also reinforces the notion of a wasteful society. The magnitude of this problem has led to increasing pressure from the public for efficient and practical measures to solve the waste problem. This paper analyses the impact of plastic use and disposal in Ghana. It emphasizes the need for commitment to proper management of the impacts of plastic waste and effective environmental management in the country. Sustainable Solid Waste Management (SSWM) is a critical problem for developing countries with regards to climate change and greenhouse gas emission, and also the general wellbeing of the populace. Key themes of this paper are producer responsibility and management of products at end of life. The paper proposes two theatrical recovery models that can be used to address the issue of sachet waste in Ghana.

## 1. Introduction 

Ever since the publication of *The Limits to Growth*, it has been broadly acknowledged that environmental sustainability cannot be achieved without altering the current system of mass consumption [1,2,3]. Problems with waste management are a concrete manifestation of the impacts of consumption patterns, and it has been suggested that seeking a solution for the waste problem could provide a key to breaking the impasse, as it forces society to think about its flow of materials [3,4]. The choice of waste management methods depends on several factors including the waste stream, equipment capacity and finance. Sustainable solid waste management is a crucial problem not only for developing countries but also for the developed countries. However, what differentiates their effectiveness in dealing with waste generated, perhaps, are the general attitude of individuals to waste, and the fact that developed countries have developed specific policies to deal with each waste stream.

In Ghana, rapid urbanization, lack of funding and economic decline in the 1970s through to the 1980s were cited as possible reasons for the poor sanitation of most towns and cities [5,6]. However, the recent upsurge in waste disposal problems can be attributed to people’s general attitudes and perceptions towards wastes [6,7]. Open, unregulated dumps are still a predominant feature of waste disposal in most parts of Ghana. The organic component of solid waste that is generated may not be too much of a problem (depending on the disposal option) as it is biodegradable, but the inorganic components are quite problematic because they are non-biodegradable and therefore can remain in the environment for a considerable length of time causing severe problems. Inorganic wastes such as plastics are scattered here and there in many communities. The management of plastic waste through incineration is not environmentally friendly and sustainable since this may release carbon dioxide, a major contributor to global warming (greenhouse effect). Landfilling plastic waste is also not desirable since plastic is non-degradable and no economic value can be derived from the waste in such a case. Although technological solutions such as sanitary landfilling, recycling, incineration and bioreactor treatment have been implemented to handle the type and quantity of waste generated in Ghana, their contributions to the effective management of waste have been insignificant and low [8,9].

## 2. Objectives of the Paper

The paper seeks to propose theoretical scenarios for sachet water waste collections, the most prominent plastic waste in Ghana. The overall objectives of this paper have been grouped into two folds:

- To investigate the actual situation of plastic waste management.
- To identify and propose a more sustainable plastic waste recovery strategy in Ghana.

The work focuses on outlining a theoretical recovery strategy to effectively manage the plastic waste (specifically sachet waste) in the packaged water production industry from a sustainability perspective balancing economic, social and environmental objectives which are frequently in conflict. 

## 3. Methods

This is a descriptive-qualitative research utilizing literature on the theme of this paper and observation of best practices. The descriptive models are based on producer responsibility studies’ literature and practical activities of waste management in enterprises managing waste in most developed countries. The study was pursued both as a literature study and based on reports from the package water manufacturing industry and informal recovery/recycling businesses, as well as involving legislative and governmental institutions. Scientific papers served as a major source of data especially in conceptualising the theme of the topic.

## 4. Generation of Plastic Waste and Management in Ghana

Over the last few decades, there has been a steady increase in the use of plastic products resulting in a proportionate rise in plastic waste in solid waste streams in large cities in sub-Sahara Africa [4,10,11,12]. Plastics are used extensively in both food and water packaging industries because of their inherent properties such as low bulk densities and inertness which make them convenient carrier materials with low risk of contamination. Plastic bottles and sachets used to package water have become widespread in the sub-region. The prominent plastic materials in commerce across the sub-region include low-density polyethylene (LDPE) commonly called polyethylene films, high-density polyethylene (HDPE) and other plastics such as polypropylene, polystyrene, polyvinyl chloride (PVC) and polyethylene terephthalate (PET). The share of plastic waste in municipal waste in Ghana has been increasing over the years (as seen Table 1). 

**Table 1:** Percentage of plastic waste in municipal waste over the years.

YEAR	% of Plastic waste
1979	1.4
1993	4
1997	5
2000	8

Note: Source: [10,13,14,15,16].

In 1979, the percentage was 1.4%, which rose to 4% in 1993, in 1996/1997 the proportion of plastic waste was 5% and by 1999/2000 its proportion increased to 8%. The per capita generation of plastic wastes stands at 0.016**–**0.035 kg/person/day, and plastics make up between 8% and 9% of the component materials in the waste stream [14]. 

The continual increase of the share of plastic waste among solid waste is a result of the huge demand for plastic products in the country, propelling private enterprises to commit huge capital to the plastic industry. By 1996, there were about 20 plastic producing establishments in Ghana and by the turn of the century there were about 40 plastic manufacturing companies producing about 26,000 metric tons of assorted plastic products annually, with 90% of these companies in the Kumasi and Accra-Tema Metropolitan Areas. Additionally, over 10,000 metric tons of finished plastic products are imported annually into Ghana [13,16]. 

### 4.1. Sachet Water Use and Environmental Impacts

The packaged water industry is characterised by both small and medium to large scale industries that pack and machine-seal sachet water and also offer bottled water to consumers. Packaged water has become the preferred mode of drinking water both at home and in public. Bottled and sachet water is available in different sizes to satisfy different consumer needs. The bottles water sizes commonly available are 0.5 L, 0.75 L, 1 L and 1.5 L and for sachet water the available quantities are 500 mL and 200 mL sachets. However, the 500 mL sachet is most common on the market. This water is referred to as “pure water” by many of the locals. The sachet water industry in Ghana is a vibrant and highly profitable sector since there is always ready market demand for its products. 

The small scale sachet water companies usually produce between 15,000 sachets (500 bags) and 45,000 (1500 bags) sachets per day and have much smaller distribution coverage, more often distributing their products in and around the towns/communities where their factories are located. Usually each bag contains 30 sachets of 500 mL of water. The medium scale water companies have the technology, resources and adequate logistics support to produce both bottled and sachet water for more than one town and even may be able to cover a district. The large scale water companies produce both bottled and sachet water for sale nationwide, and most operate water packaging factories and depots in several towns and cities across Ghana. They usually produce and supply over 5000 bags of sachet water per day. There has been a proliferation of sachet water producing companies all across the country due to the relatively low start-up capital required. 

Regardless of the accruing benefits like access to portable drinking water and employment generation through sachet water trade, due to sachet water production and consumption, the indiscriminate disposal of the waste in various undesired sites such as along the streets, gutters, motor parks, schools, markets, homes, and venues of social functions *etc*. poses many environmental threats. The sachets are made of non-biodegradable synthetic polyethylene (polythene) which does not decompose in the soil even after many years. The polythene even when subjected to burning produces major known and harmful greenhouse gases (GHGs) like carbon monoxide, nitrous oxide and carbon dioxide. 

### 4.2. Sachet Waste Management and the Informal Sector

An informal waste recycling activity is a phenomenon in developing and least developed countries as a result of low economic development [17]. There are at least four main categories of informal sachet waste recovery and recycling activities, and categories are stated as follows [17,18]:

- Itinerant waste buyers: these are waste collectors that are engaged in the collection and marketing of sorted dry recyclable materials. They collect the recyclable items from door to door, and this category of informal waste collectors are common is most parts of the world.
- Street waste picking: recyclables are recovered from mixed waste on the streets or from communal bins.
- Municipal waste collection crew: recyclables are recovered from vehicles transporting Municipal Solid Waste to the dump site.
- Waste picking from dumps: these are waste collectors that recover recyclables from dump sites before being covered.

In Ghana, the informal collection and recycling sector is an important, but often unrecognised, part of the solid waste and resources management system, and it is estimated that about 20%–30% of recycling is achieved by way of informal recycling systems, reducing collection and disposal costs. They play a vital role in the value chain by reprocessing waste into secondary raw materials, providing a livelihood to a greater number of urban populations.

As the issues of plastic waste have gained much needed attention over the years, a task force was set up in the early 2000s with the agenda of turning plastic waste into a commodity for trade. The task force embarked on a series of campaigns to sell the idea of waste as a commodity to Ghanaians. The campaign caught on very well as Ghanaians enthusiastically started collecting plastic waste, especially sachet water waste for sale even though most of the collection was done by the informal sector and usually at the dumpsite or landfills. The task force however managed to promote the business by setting up plastic waste collection points mostly in the urban areas where individuals could send their plastic for sale. Some of the collectors of the plastic waste banded together to form the Plastic Waste Collectors Association of Ghana (PLWAG). The task force was gradually phased out due to financial reasons. As the task force was phased out, the government co threatened to ban plastic waste generating products which led to the birth of *Accra Plastic Waste Management Project* by the private sector, mainly consisting of sachet water producers and sellers, as well as plastic manufacturers in December 2007. The major feature of the project was that, in addition to generating funds to manage the plastic waste in the capital, the project had the power to prosecute littering offenders according to the Accra Metropolitan Assembly’s by-laws, was able to recruit plastic waste guards as well as educate the general populace on the appropriate disposal of plastic waste. 

The threat by government to ban plastic production completely if carried out will increase the cost of production of packaged water and/or worsen the unemployment situation in the country. The national association of sachet water producers recognises the impact and environmental concerns of indiscriminate disposal of wastes from the consumption of their products. In this regard, there have been a number of programmes and efforts on their part to help curtail the sachet water waste threat in Ghana. Such programmes include the provision of bins in public places to facilitate the disposal of sachet wastes, assisting in the transfer of technology to members, facilitating training courses in bookkeeping and environmental education. 

There are also some NGOs like Trashy Bags which turns sachet and other plastic wastes into reusable shopping bags, fashion accessories, school supplies, and other products. The company employs about 60 Ghanaian workers to collect, clean and stitch plastic trash in the form of sachets that contain water and other beverages [19,20,21,22].

## 5. Theoretical Framework for Sachet Waste Recovery

The main aim of the strategy is to establish a community based approach to sachet waster waste management in which responsibilities are shared between households, the city authorities and producers of the sachet water. By encouraging producer responsibility and household participation, a greater portion of the sachet water waste, which usually ends up at dump sites, can be recovered efficiently and at low cost. The programme will seek to functionalize a multi-stakeholder return and/or buy back system that will facilitate the collection and return for recycling of sachet waste that normally finds its way into our environment. The concepts of the programme are discussed below.

### 5.1. Setting the Goals of the Recovery System

There are different ways of implementing a recovery system to achieve a desirable goal. The main goal is to increase collection and recycling of sachet waste of products at the end of life and also to encourage producers to be more environmentally responsible.

### 5.2. Collection Methods for Sachet Waste

#### 5.2.1. Deposit-Refund System

It is a system where the consumers’ deposits will be refunded when handing in the used product. Here, the consumer receives a financial compensation when returning a discarded product which corresponds to a specified deposit paid when purchasing the product. Deposit-refund systems can be divided into natural and artificial systems. Natural systems are where the real value of the container induces the desire of producers to recover them. The refunds on such products have to be high enough to motivate consumers to return them after end of use, instead of keeping them for their own purposes or throwing them away. Deposit-refund systems are in many instances seen as the best solution when very high collection rates are desired. In Europe, for instance, many of the traditional deposit-refund systems for beer and soft drinks in refillable glass bottles are claimed, where they still exist, to lead to an almost 100% return rate.

#### 5.2.2. Kerbside Collection System

It is a system where the discarded products are collected in close proximity to consumers or households similar to the way the ordinary household waste is collected. The large-scale kerbside collection system is the German packaging collection is a great example of the potential of achieving high collection rates in this system. Consumers may drop off sachet waste at collection points that are convenient and of easy access to encourage maximum collection. 

#### 5.2.3. Bring System

It is a system where the consumer is expected to bring the discarded products to a container or something similar, which is placed at a shorter or longer distance from the home of the consumer. These systems include drop-off centres and recycling stations, among other things. The packaging waste collection as organized in Sweden is an example of a system that mainly relies on the consumers to bring the discarded products to containers, which are distributed in various parts of the cities. The collection results are fairly mixed but this system could be used to recover most of the sachet waste in Ghana.

### 5.3. Implementation of Sachet Waste Recovery

Extended producer responsibility is a strategy designed to promote the integration of environmental costs associated with goods throughout their life cycles into the market price of the products [23]. There are a number of instruments that can be used to shift responsibility for managing product and packaging waste from government and taxpayers to producers and consumers. These include regulatory instruments such as mandatory take-back schemes, minimum recycled content standards, materials and product bans and restrictions, economic instruments including advance disposal fees, virgin material levies and deposit/refund systems. Responsibility can be narrowed down to mandatory financial responsibility for the producer with the option of voluntary physical responsibility. 

The concept was first formally introduced in Sweden by Thomas Lindquist in a 1990 report to the Swedish Ministry of the Environment [24]. In this report, Thomas Lindhqvist specifies how producers can take responsibility for their products by distinguishing between four different forms of responsibility [25]:

- ***Liability***: refers to a responsibility for proven environmental damages caused by the product in question. The extent of the liability is determined by legislation and may embrace different parts of the life-cycle of the product, including usage and final disposal.
- ***Economic responsibility***: means the producer will cover all or part of the costs for collection, recycling, or final disposal of the product manufactured. These costs could be paid directly by the producer or by way of a special fee.
- ***Physical responsibility***: is used to characterize the systems where the producer is involved in the actual physical management of the products or the effects of the products.
- ***Informative responsibility***: signifies several different possibilities to extend responsibility for the products by requiring producers to supply information on the environmental properties of the products manufactured.

### 5.4. Scenario 1; Sachet Water Producer Recovery Programme

**Figure 1:**
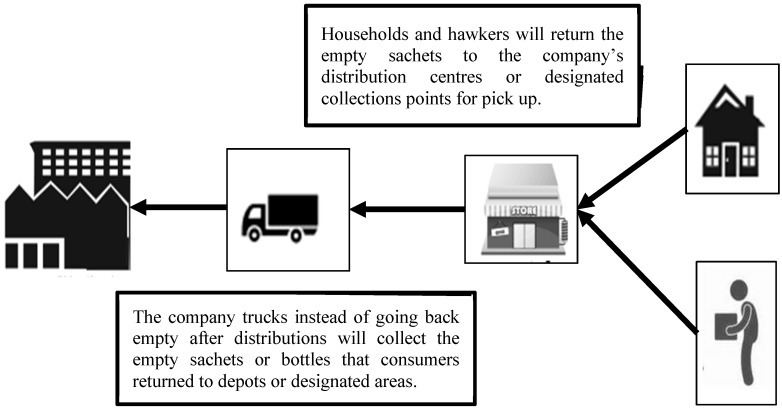
Producer recovery model for sachet waste.

The producer recovery model as shown in Figure 1 refers to the sachet water manufacturing enterprises and retailers of plastic products, establishing their own sachet waste recovery systems by assuming physical and financial responsibilities for the end of life management of their products. The model can be used by small to large scale sachet water producers as well as industrial plastic producers. Sachet/bottled water companies in Ghana could establish a wide range of reverse logistics network for the recovery of sachet waste.

Producers of sachet or bottled water can receive back plastic waste by providing incentives like discount coupons, the plastic waste they in turn return to plastic producers based on similar incentives or sell them to recycling companies for processing. Such a recovery system invokes a kind of extended producer responsibility. In Figure 1 below, sachet water manufacturers can recover the sachets from consumers and retailers. The cost of collection and transportation can be recovered by using their distribution trucks to pick up the sachets on route back after distribution instead of going back empty handed. They can then sell the collected sachets to plastic recycling firms which can reduce costs and in turn eliminate some of this plastic waste which might end up in landfill sites, or worse, litter the environment as is the case currently.

Also, instead of the producers taking back the sachets from consumers themselves, they can partner with organised informal waste operators who will be tasked specifically to collect or recover the sachet waste before it ends up at landfill sites. They can invest in developing the needed skills and techniques among these informal operators.

### 5.5. Scenario 2; the State Run Recovery Programme

**Figure 2:**
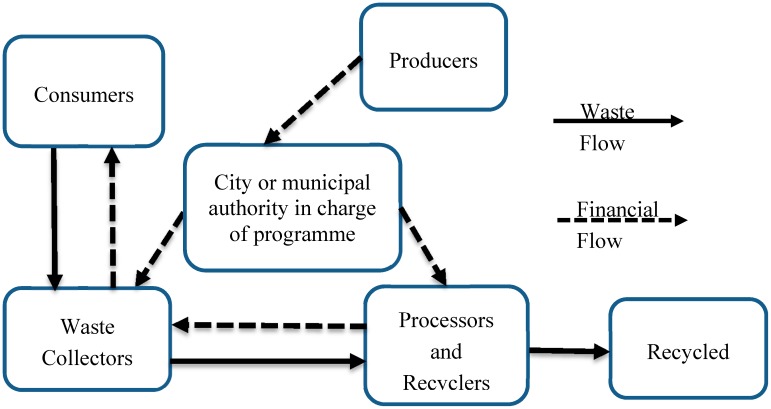
State or government run recovery system model.

As shown in Figure 2, the municipal or state is the main body of public investment in the establishment of sachet waste recovery and recycling programs. The state or municipal authorities will have the physical responsibility of collecting the sachet waste while the producers will be financially responsible for the programme and also providing incentives to consumers to encourage return of sachet waste to vantage points or collections points provided by the municipal authorities. This can also be done for other useful waste streams instead of them ending up at landfill sites or being disposed of improperly. 

### 5.6. Waste Flow

Consumers (including households and businesses) bring post-use plastic products to collection points or to waste collectors (formal or informal), who then transport it to processors or recycling firms. The collections can be coordinated by the authorities, producers or a body in charge of the program for maximum efficiency, and this can be done by integrating and improving on the already existing informal recovery systems. 

### 5.7. Financial Flows

Each producer pays a financial contribution to the collection scheme. The amount paid, for instance, can depend on the amount of production of each producer. These payments are then disbursed by the authority in charge of the scheme to the actors in the collection process. The authority pays each collector based on a unit rate per weight that they handle, as already existing in the informal sector, or the collectors can sell their products to the recycling companies.

## 6. Stakeholder Participation, Public Education and Awareness

Solid waste management is basically a welfare and development matter and it is commonly accepted that public participation is essential for its success. Stakeholder participation entails the involvement of all groups of people in the identification of their felt needs, mobilization of resources, and deciding on the direction and execution of programs and projects. It should take place at all levels of planning and management, including training, problem identification, implementation, monitoring and evaluation. Awareness, on the other hand, is the process of awakening and raising people’s sensitivity to concerns, in this case the plastic waste management problem in Ghana.

Awareness should be created through formal and non-formal education with the assistance of both the print and electronic media. Environmental education with respect to plastic waste management, formal and non-formal, is vital to changing people’s attitudes to appreciating a clean and safe environment, and leads to their empowerment in enabling them to manage their wastes sustainably. It also creates responsibility among the different communities, increases environmental accountability and governance and encourages the rational use of environmental resources. There is need to create a mechanism for stakeholder participation and dialogue so as to empower and enable the public to participate in sound environmental practices. This may include:

- Coordinating the preparation and distribution of information about the strategy and on how to innovatively manage plastic wastes in Ghana.
- Coordinating the preparation and presentation of weekly radio and TV programs on the environmental impacts of indiscriminate dumping and littering of the urban environment with plastic litter; Commissioning of the preparation and publication of bi-monthly feature articles in the print media on environmentally sound strategies of dealing with plastic wastes.
- Coordination of the holding of public meetings in all divisions to sensitize the people on the negative impacts of plastic waste management and the need to have then reused and recycled.

## 7. Conclusions

The ineffective tax levy systems, lack of incentives for the people of Ghana to separate waste, coupled with the overall negative attitude require that Ghana considers an approach that will involve all stakeholders in the plastic pollution problem. Policy approaches like product stewardship and extended producer responsibilities, if implemented right, can assist government in shifting responsibilities for the management of certain waste streams from the state to the producers, thereby reducing costs. Product stewardship is usually designed as a voluntary system that shares responsibility for the adverse environmental effects of products by all parties involved in the lifecycle whereas EPR focuses all the responsibility for waste management onto manufacturers [26,27,28,29].

A community based approach to sachet waste management in which responsibilities are shared between households, city and municipal authorities and producers of the sachet water is needed. Through producer responsibilities and community participations, a greater portion of the sachet water waste, which usually ends up at dump sites and the environment, can be recovered efficiently and at low cost. 

The success of recycling not only depends on participation levels, efficiency of the equipment and infrastructure but on the quality of recovered waste. Therefore, it is necessary to recover recyclables at the early stage before they are mixed with other waste streams or end up at landfills. 

### 7.1. Improving the Informal Waste Plastics Recovery Sector 

As steps are taken to establish a formal recovery sector, it is necessary to intensify efforts towards rebuilding the formal sector since the operating costs for collecting and recycling in the informal sector are much lower. However, there are serious threats to individual operators in recovery of the recyclable waste as their operations are not done under ideal conditions. Most informal waste sector workers (dump and street waste pickers) constitute the lowest level of society, even though their income and living conditions differ significantly according to their main activities, working conditions are unimaginable and include permanent exposure to dangerous, toxic and contagious substances. Therefore, the government should develop a management system which integrates the operations of the informal sector and also assists in improving their conditions. Creating a more positive public and political attitude towards waste pickers through civil society campaigns promotes informal sector integration.

Since proper waste management is not the sole responsibility of the government, increasing the sense of responsibility of industries—like the packaged water industry—for their product impacts is necessary, such as where the packaged water producers finance a recovery programme. The packaged water industry, for example, can engage the informal waste sector to recover the waste from their products. The economic and environmental benefits of such cooperation are obvious. 

### 7.2. Expanding the Capacity of the Plastics Recycling in Ghana

Political will as well as attitude change is important in expanding the national recycling capacity. It is a matter of formulating national policies, respective laws and regulations for better collection and sorting of specific waste streams, limiting or banning landfilling of waste streams like plastic. Also, government should assist by removing obstacles to the entrepreneurial activities of both informal sector operators and organisations, which is an important factor in sustainable waste management. Alternatively, the government should promote research and development (R&D) focusing on present recycling technologies (*i.e.*, pre-treatment stage), new forms of recycling technologies such as chemical recycling, and the recyclability of end-products.
